# Oral *Eikenella* as a potential new biomarker of symptomatic carotid atherosclerosis

**DOI:** 10.1080/20002297.2026.2613521

**Published:** 2026-01-12

**Authors:** Kristine Stø, Karolina R. Skagen, Kristian Holm, Thor Ueland, Beate Vestad, Vigdis Bjerkeli, Bente Halvorsen, Marius Trøseid, Johannes R. Hov, Mona Skjelland

**Affiliations:** a Institute of Clinical Medicine, University of Oslo, Oslo, Norway; b Department of Neurology, Oslo University Hospital, Oslo, Norway; c Research Institute of Internal Medicine, Oslo University Hospital, Oslo, Norway; d Section of Gastroenterology, Department of Transplantation Medicine, Oslo University Hospital, Oslo, Norway; e Section of Clinical Immunology and Infectious Diseases, Oslo University Hospital, Oslo, Norway; f Norwegian PSC Research Center, Department of Transplantation Medicine, Oslo University Hospital, Oslo, Norway

**Keywords:** Oral microbiota, carotid atherosclerosis, cerebrovascular disease, biomarkers, inflammation, *Eikenella*

## Abstract

**Introduction:**

Oral microbiota dysbiosis is linked to cardiovascular disease, and oral pathogens have been detected in atherosclerotic plaques. We aimed to investigate the relationship between the oral microbiota and carotid atherosclerosis, and the occurrence of oral pathogens in plaques.

**Patients and methods:**

Oral swab and saliva samples from patients with severe carotid atherosclerosis (≥50% stenosis) were compared with those from controls. The oral microbiome was analyzed by 16S rRNA amplicon sequencing targeting the V3‒V4 region. Carotid plaques were investigated for five oral bacterial species by qRT-PCR.

**Results:**

Compared with controls, patients exhibited different inter-individual (beta) diversity (*r* = 0.02, *p* = 0.002), reduced intra-individual (alpha) diversity (*p* = 0.026) and 22 bacterial genera differed in relative abundance. Furthermore, abundances of five bacterial genera, including *Eikenella,* were increased in patients with recent cerebrovascular symptoms compared to asymptomatic patients. *Eikenella corrodens* was detected in all 30 carotid plaques.

**Conclusion:**

Oral microbiota diversity and composition differ between patients with carotid atherosclerosis and controls. A higher relative abundance of the genus *Eikenella* in symptomatic versus asymptomatic patients and the detection of the species *Eikenella corrodens* in all carotid plaques, might suggest that *Eikenella* is important in atherogenesis and plaque instability. Oral *Eikenella* could possibly serve as a potential new biomarker.

## KEY MESSAGES

- Oral *Eikenella* was increased in patients with symptomatic carotid atherosclerosis.
- *Eikenella corrodens* was detected in all 30 carotid plaque specimens.
- Oral *Eikenella* might serve as a new biomarker for atherogenesis and plaque instability.

## Introduction

Evidence suggests that an imbalance of the human microbiota, primarily in the gut – i.e. dysbiosis, plays a role in cardiovascular disease, including atherosclerosis [1]. Less is known about the oral microbiota and its role in systemic disease. Several studies have shown associations between periodontitis and ischemic stroke [2,3]. Oral microbiota dysbiosis is directly linked to the development of oral mucosal disease and periodontitis, which are associated with systemic low-grade inflammation, chronic arterial inflammation and an increased risk of cardiovascular diseases [4]. A recent study of 869 participants investigated the oral microbiome and carotid intima-media thickness (cIMT) and found that changes in oral microbiota diversity, as well as oral bacterial genera, were linked to cIMT in males but not in females [5].

Several species of oral bacteria that cause periodontitis and dental caries have been found in atherosclerotic plaques [6–9]. In a study of 51 patients with periodontitis and coronary artery disease, four common periodontal pathogens were identified in coronary plaques [9]. Similarly, in a study of 42 atheromatous plaques from carotid endarterectomies, periodontitis-associated bacteria were identified in all samples, with the simultaneous presence of several bacterial species in most specimens [8].

It has therefore been proposed that periodontal bacteria can enter the systemic circulation and stimulate the production of inflammatory factors, leading to vascular endothelial dysfunction and the formation of atherosclerotic plaques [10]. Oral bacteria may release arginine-specific gingival proteases to promote the activation of platelets and lipid deposition, ultimately accelerating atherosclerosis [11,12]. *Fusobacterium nucleatum* and *Porphyromonas gingivalis* are common periodontal pathogens that have been demonstrated to aggravate chronic inflammation [13]. Imbalances of oral commensal nitrate-reducing bacteria have been associated with a decreased level of nitric oxide, causing endothelial dysfunction [14].

We previously assessed the gut microbiota in patients with carotid atherosclerosis and healthy controls [15] without showing differences in diversity or composition. However, we found increased levels of the short-chain acid butyric acid in patients.

In this relatively unexplored field of oral microbiota, we hypothesized that patients with carotid atherosclerosis would have a different oral microbiota than healthy controls, that oral microbiota might differ between symptomatic and asymptomatic patients, and that oral bacteria would be detected in carotid plaques. The present study aims to expand knowledge about the association between the oral microbiota and carotid atherosclerosis by identifying oral microbiota signatures associated with symptomatic and asymptomatic plaques, as well as detecting oral bacteria in carotid plaques.

## Patients and methods

### Study population

The study population has been described previously [15]. In brief, this cross-sectional observational study was conducted between August 2017 and June 2019. Sixty adult patients with severe carotid atherosclerosis were included and compared to 44 healthy controls. Seventy-eight patients provided fecal and oral samples available for 16S rRNA sequencing analysis. Thirty patients scheduled for carotid endarterectomy provided oral samples and were available for qRT-PCR analysis, as shown in Supplementary Figure 1. The study was approved by the Norwegian Regional Committees for Medical and Health Research Ethics (ID REC 2017/2202 A) and was performed in accordance with the Declaration of Helsinki. All the participants gave written informed consent before inclusion.

### General health, diet, blood sampling and carotid ultrasound

Information on general health and diet was collected from questionnaires and medical journals. Blood sampling was performed in a fasting state, and bilateral carotid ultrasound was performed at inclusion [15].

### Oral sampling and storage

Oral samples were collected in a fasting state, i.e. no food/drinks (water allowed), tobacco, snuff, chewing gum or tooth brushing since the previous night. Unprovoked saliva (3 mL) was collected in sterile tubes without additives. Using a dry sterile cotton bud, the inner and outer surfaces of the upper and lower tooth rows and the back of the tongue were swabbed. The samples were frozen at −80 °C within 10 min.

### Carotid plaque sampling and storage

Carotid plaques removed en bloc during surgery were stored immediately in Allprotect (Qiagen, Hilden, Germany) at room temperature for up to 14 days before being frozen at −80 °C until further analysis.

### Analysis of oral microbiota by 16 s rRNA sequencing analysis

Oral DNA was extracted using the commercial ZymoBIOMICS™ DNA Miniprep kit (ZR, Zymo Research, Irvine, CA, USA), according to the manufacturer's instructions, with slight modifications.

Libraries for 16S rRNA amplicon sequencing were generated as previously described [16]. Paired-end reads were filtered for Illumina Universal Adapters and PhiX, demultiplexed, quality trimmed and merged using BBDuk 39.01, Cutadapt 4.4 and BBMerge 39.01 [17–19]. Denoising to ASVs, taxonomic classification, filtering of contaminants and rare ASVs, and building of a phylogenetic tree were performed with QIIME2 version 2023.5 [20] and the Silva database version 138.1 [21]. The contaminants were filtered with the R package microDecon [22]. To reduce the effect of uneven sequencing depths, samples were rarefied to an even level of 7086 counts per sample, and all diversity analyses were performed on this rarefied dataset. Differential abundance testing with ANCOM-BC2 was performed on a prevalence-filtered (10%) version of the non-rarefied dataset. Alpha and beta diversity metrics were calculated in QIIME2. A dysbiosis index for oral swab samples in carotid atherosclerosis was calculated (formula provided in the Supplementary material).

### Analysis of oral and carotid plaque samples by quantitative real-time PCR (qRT-PCR)

Carotid plaques were pulverized using a Cysolys Evolution (Bertin Instruments, Montigny-le-Bretonneux, France). DNA extraction was performed using the commercial ZymoBIOMICS™ DNA Miniprep Kit (ZR, Zymo Research, Irvine, CA, USA), according to the manufacturer's instructions, with slight modifications. The extracted DNA concentration and purity were determined using a NanoDrop 2000 spectrophotometer.

DNA from oral swabs and carotid plaques was used for quantification of the bacterial species *Eikenella corrodens, Fusobacterium nucleatum, Porphyromonas gingivalis, Aggregatibacter actinomycetemcomitans*, *Treponema denticola* and the universal primer 16S rRNA by qRT-PCR. Primers were designed by Customer Services, Merck Life Science, UK (Supplementary Table 1).

All PCR assays were carried out in duplicate. Reagents concentrations were adjusted to comply with a final reaction volume of 20  µL, containing 100 ng of template DNA, 200 nM of sense and antisense primers and PerfeCTa® A SYBR® Green FastMix® Reaction Mix (Quantabio, Beverly, MA, USA). The negative control was sterile distilled water instead of template DNA.

The reaction components were dispensed into a 96-well assay plate and cycled in a Stratagene® Mx3005 Detector (Stratagene, now Agilent, Santa Clara, CA, USA). The reactions were carried out in a Step-One real-time thermocycler/detector for 40 cycles. The Mx3005 Detector software calculates a Ct value that is proportional to the number of gene copies in the original sample.

Raw Ct values (quantification cycle) obtained from the qRT-PCR were used for the analysis, and the mean Ct value of each biological replicate was normalized to the 16S rRNA 'housekeeping' gene, generating a relative quantity for each target organism. Relative quantities were calculated using the ΔCt method (Target gene Ct—16S rRNA Ct), providing a measure of the bacterial load relative to the bacterial DNA in the sample. Samples with Ct values above 35 cycles were considered undetectable and assigned a value of zero for analysis [7,23].

### Statistics

Descriptive statistics are given as number and proportion (%), mean with standard deviation or median (min–max). The Mann‒Whitney U test was used to compare the non-parametric categorical variables with continuous variables. The Spearman’s rank correlation test was used to evaluate relationships between variables.

*p*-values are two-sided and considered significant when <0.05. IBM SPSS Statistics for Windows, Statistical Software version 25.0 (IBM Corp., Armonk, NY, USA) was used for data analyses.

## Results

### Baseline characteristics of patients and healthy control subjects

For the oral 16S rRNA sequencing analysis, a total of 76 saliva samples and 69 oral swab samples from 78 participants were collected (Supplementary Figure 1). The demographic and clinical characteristics are presented in Table 1. Patients were older than controls and had more risk factors for stroke, as reflected by their hypertension, dyslipidemia, and diabetes, as well as higher waist–hip ratio, C-reactive protein (CRP) and leukocyte counts. Patients had lower cholesterol levels (both LDL and HDL) than controls, probably related to the use of statins. Whereas none of the controls used antibiotics in the last three months, 19.5% of the patients reported using antibiotics, but none used antibiotics during the last three weeks.

**Table 1.** Baseline characteristics of patients and controls.

	Patients *N* = 41	Controls *N* = 37	*p*-Value
Age (years)*	72.3 (6.4)	66.8 (4.9)	0.001
Male sex	43.9 (18)	27.0 (10)	0.121
Waist-hip ratio (cm/cm)	0.95 (0.08)	0.89 (0.07)	0.001
Hypertension	78.0 (32)	21.6 (8)	<0.001
Anti-platelet treatment	80.5 (33)	8.1 (3)	<0.001
Statin treatment	78.0 (32)	10.8 (4)	<0.001
C-reactive protein (mg/L)*	3.5 (5.3)	1.2 (0.98)	0.014
Leukocyte count (10^9^/L)*	7.8 (1.9)	5.1 (1.1)	<0.001
Total cholesterol (mM)*	4.2 (0.98)	5.3 (0.27)	<0.001
LDL cholesterol (mM)*	2.3 (0.86)	3.2 (0.85)	<0.001
HbA1c (%)*	5.7 (0.87)	5.3 (0.28)	0.006
Antibiotics last 3 months	19.5 (8)	0 (0)	0.005
Current smoker	12.2 (5)	5.4 (2)	0.295

Values are given as % (*n*) or *mean (SD).

We further compared symptomatic patients (i.e. ischemic stroke or transitoric ischemic attack (TIA), within the last three months) with asymptomatic patients. Baseline characteristics are shown in Table 2. The groups were similar, but symptomatic patients had a higher waist‒hip ratio and leukocyte counts. There was no difference in the degree of stenosis, but more non-calcified plaques in the symptomatic group.

**Table 2.** Baseline characteristics of asymptomatic and symptomatic patients.

	Symptomatic patients *N* = 15	Asymptomatic patients *N* = 26	*p*-Value
Age (years)*	73.6 (7.0)	71.5 (5.9)	0.327
Male sex	60 (9)	34.6 (9)	0.115
Waist-hip ratio (cm/cm)	1.0 (0.07)	0.93 (0.07)	0.001
Hypertension	86.7 (13)	73.1 (19)	0.311
Anti-platelet treatment	100 (15)	69.2 (18)	0.017
Statin treatment	86.7 (13)	73.1 (19)	0.311
C-reactive protein (mg/L)*	3.9 (5.1)	3.1 (5.4)	0.757
Leukocyte count (10^9^/L)*	8.6 (1.7)	7.3 (1.9)	0.043
Total cholesterol (mM)*	3.7 (0.91)	4.4 (0.93)	0.030
LDL cholesterol (mM)*	2.2 (0.79)	2.3 (0.90)	0.730
HbA1c (%)*	5.8 (1.2)	5.7 (0.77)	0.837
Antibiotics last 3 months	25.0 (4)	18.5 (5)	0.952
Current smoker	13.3 (2)	11.5 (3)	0.866
>70% stenosis	80 (12)	73.1 (19)	0.273
Non-calcified plaques	66.7 (10)	23.1 (6)	0.003

Values are given as % (n) or *mean (SD).

Among the 30 patients who underwent carotid endarterectomy, 17 were also included in the oral 16S analysis. All provided oral swab samples. Twenty-five patients were symptomatic within the last 1.5 months, 21 had MRI scans showing acute cerebral infarctions, one had a small stroke 5.5 months ago, whereas four had unspecific symptoms.

### Oral microbiota composition overview and correlation between oral swabs samples and saliva

**Figure 1.**
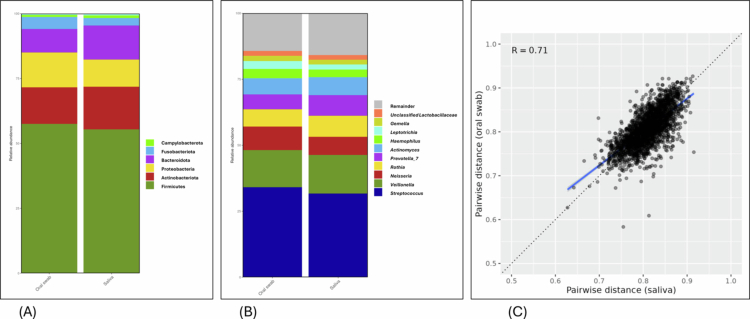
Microbiota composition in oral swabs and saliva in the total study population. Microbiota composition at the phylum level in oral swabs (left column) and saliva in the total study population (A), and at the genus level (B) in the total study population. The Mantel test shows good correlation between saliva (x-axis) and oral swab samples (Jaccard distances). *R* = 0.71, *p* = 0.001 (C).

The final oral microbiota analysis was comprising 6,396,158 reads, with a minimum read count of 7,086 per sample. The most abundant phylum was Firmicutes, accounting for more than 50% in both sample types, followed by Actinobacteria, Proteobacteria, Bacteriodota, Fusobacteria and Patescibacteria (Figure 1A). The ten most abundant genera were S*treptococcus*, *Veillonella, Prevotella_7, Rothia*, *Neisseria*, *Actinomyces, Haemophilus, Leptotrichia, Gemellas and Prevotella* (Figure 1B). The overall microbiota composition in saliva and oral swabs was distinct but closely correlated (Jaccard distances, *r* = 0.71, *p* = 0.001) (Figure 1C). In the further analysis, we focused mainly on oral swabs, as both collection and DNA extraction were found easier.

### Oral microbiota in patients with carotid atherosclerosis versus healthy controls

**Figure 2.**
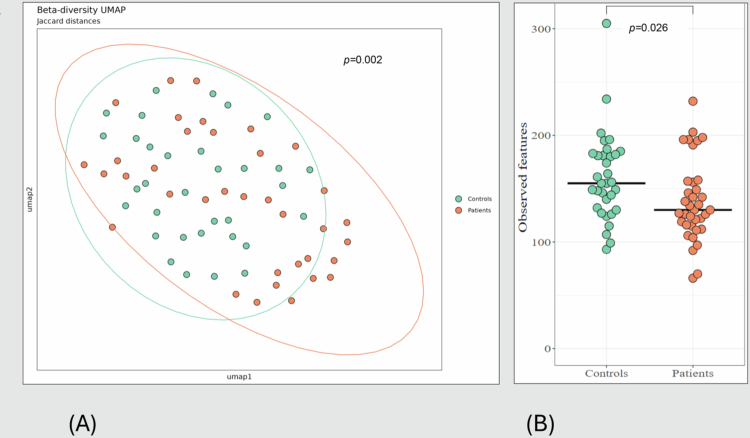
Oral microbiota in patients compared to healthy controls. Oral microbiota in patients compared to healthy controls shows significant differences in Beta diversity in oral swabs in patients (red) and controls measured by Jaccard distances (A), and the alpha diversity measured by the observed features in oral swabs in controls (green) and patients shows lower alpha diversity in patients (B).

Considering the inter-individual (beta) diversity, we detected small but significant differences between all patients compared to controls, as measured by Jaccard distances (*r* = 0.02, *p* = 0.002) (Figure 2A) and unweighted UniFrac (*r* = 0.03, *p* = 0.004). In terms of intra-individual (alpha) diversity, this was reduced in patients, as measured by the number of observed features (*p* = 0.026, adjusted for sex, age and BMI) (Figure 2B). Phylogenic diversity measured by the Faith PD index, also showed significant differences (*p* = 0.005).

After filtering out bacterial genera present in less than 10% of the samples, with a false discovery rate (FDR)  <0.1 and log2fc >1, 147 genera were included in the analysis. We found that 22 genera differed significantly between patients and controls, with 12 genera from four phyla being higher in patients than controls (including *Lactobacillus*), whereas ten genera from four phyla were higher in controls (Supplementary Table 2).

Next, a dysbiosis index for carotid atherosclerosis was calculated based on the up- and downregulated genera described above (see Methods). The dysbiosis index was negatively correlated to alpha diversity (observed features) (*r* = −0.28, *p* = 0.020).

### Oral microbiota in patients with symptomatic versus asymptomatic carotid atherosclerosis

**Figure 3.**
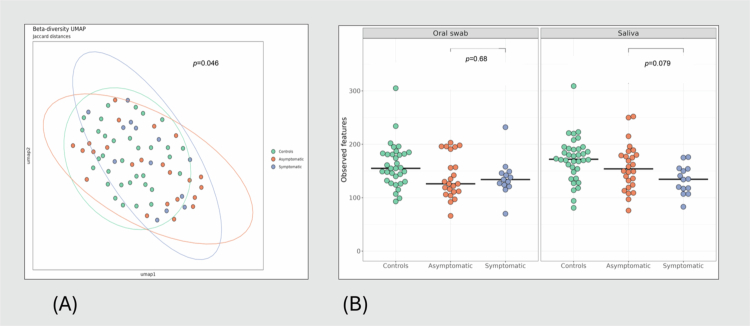
The oral microbiota in patients with asymptomatic versus symptomatic carotid atherosclerosis. Beta diversity (Bray–Curtis) shows a significant difference between asymptomatic and symptomatic patients (A), and a tendency towards a lower alpha diversity in symptomatic patients in saliva (right violin plots), but not in oral swabs (B).

When comparing patients with symptomatic versus asymptomatic atherosclerosis, there were only minor differences. We found a significant difference in one beta diversity measure in oral swabs (Bray–Curtis, *r* = 0.05, *p* = 0.046) (Figure 3A), as well as a tendency towards lower alpha diversity in saliva in symptomatic patients (*p* = 0.079, Figure 3B), but not in swabs. When comparing composition, five genera (*Saccharimonadaceae, Abiotrophia, Lautrophtia, Actinobacillus* and *Eikenella*) had a higher abundance in symptomatic patients, as shown in Supplementary Table 3.

### Associations between oral microbiota, clinical features, drugs and biochemical markers

There were few correlations overall for alpha diversity and the dysbiosis index, as shown in Supplementary Table 3. In the patient group, alpha diversity was significantly lower in smokers (12.2%) compared to non-smokers (*p* = 0.025). There was a non-significant tendency towards lower alpha diversity in patients exposed to antibiotics (*p* = 0.11). Considering plaque echogenicity, a lower dysbiosis index was associated with non-calcified plaques (*p* = 0.031).

The *Eikenella* genus (higher in symptomatic patients) was more abundant in hypertensive patients compared to non-hypertensive patients (*p* = 0.036). For the common periodontal pathogens *Fusobacterium* and *Porphyromonas,* we found no significant differences between the groups, nor any relevant correlations.

### Carotid plaque microbiota and relation to oral samples

**Figure 4.**
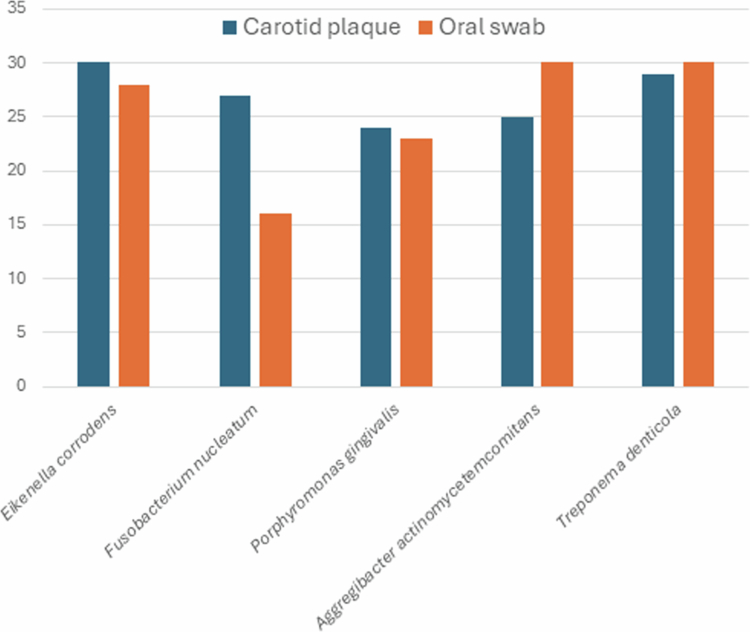
Oral bacteria in carotid plaques and oral samples by qRT-PCR. In 30 patients who underwent carotid endarterectomy, common oral pathogens were detected in all the samples. *Eikenella corrodens* was detected in 100% of the plaque samples, while the other four bacteria were detected in most plaque and oral samples.

Finally, we aimed to explore whether *Eikenella* could be detected in carotid plaques and whether the relative quantity in oral and plaque samples was correlated. We investigated the presence and relative quantity, measured by qRT-PCR for the five oral bacterial species *Eikenella corrodens, Fusobacterium nucleatum, Porphyromonas gingivalis, Aggregatibacter actinomycetemcomitans and Treponema denticola* in carotid plaques and oral samples from 30 patients. Figure 4 shows the presence of these species in carotid plaques and oral samples, respectively. We detected *Eikenella corrodens* in all (30/30) carotid samples*, Fusobacterium nucleatum* in 90% (27/30), *Porphyromonas gingivalis* in 80% (24/30), *Aggregatibacter actinomycetemcomitans* in 83.3% (25/30) and *Treponema denticola* in 96.7% (29/30). In 20 plaques, we detected all five bacteria; seven plaques had four bacteria, two had three bacteria, whereas one patient only had *Eikenella corrodens*. In oral samples, *Eikenella corrodens* was detected in 93.3% (28/30).

The relative quantities of the measured bacteria in plaques were not correlated with that in the oral samples. Moreover, we did not find other relevant associations.

## Discussion

In this study investigating the oral microbiota in patients with severe carotid atherosclerosis, as well as the presence of oral pathogens in carotid plaques, our main findings were that patients with carotid atherosclerosis had lower alpha diversity, different beta diversity and extensive differences in oral microbiota composition compared to healthy controls. In the patient group, those with recent clinical cerebrovascular events (symptomatic atherosclerosis) showed a higher relative abundance of the *Eikenella* genus, a periodontal pathogen previously described in human atherosclerotic plaques [7–9]. Furthermore, upon investigating carotid plaque samples, the *Eikenella corrodens* species was detected in all plaques (*n* = 30).

Overall, the oral microbiota was different in patients compared to controls, both of which were assessed globally with diversity measures and by the relative abundance of individual bacteria. Previous studies have shown conflicting results. Fåk [24] found a similar overall microbial structure in oral swabs between patients with carotid atherosclerosis and controls (*n* = 92), while Wang [25] reported that lower alpha diversity in oral rinse samples was associated with increased stroke risk (*n* = 60). In contrast, Sun [2] reported greater alpha diversity in the saliva of 146 stroke patients and high-risk patients. Boaden [26] did not find differences in diversity measures in 77 poststroke patients, while Kato-Kogoe [27] found significant differences in beta diversity in the saliva of patients with atherosclerotic cardiovascular disease (*n* = 129). Thus, the literature does not show uniform results, which could be related to several factors, such as study design, sample size, population characteristics (e.g. stroke, risk of stroke or atherosclerosis) and methodological variance in sampling (e.g. timing, anatomical site), storage, sequencing analysis and statistical tools. Beta diversity tends to be more often different than alpha diversity. Alpha diversity varies between anatomical sites in the oral cavity, making studies difficult to compare directly, however most have sampled unprovoked saliva. Nevertheless, our findings are consistent with the general notion that higher alpha diversity is considered a healthier microbiome, although increased alpha diversity could also reflect dysbiosis with pathogenic bacteria [25]. When compositional differences were considered, twelve bacterial genera differed between patients and controls. The *Lactobacillus* genus and genera from the *Lactobacillaceae* family differed the most. *Lactobacillus* has previously been associated with blood lipids in a similar study population [24].

Patients with symptomatic and asymptomatic carotid atherosclerosis had similar baseline characteristics, including the degree of stenosis, but symptomatic patients had more non-calcified plaques. The differences in the oral microbiota were subtle, but the genus *Eikenella* had a higher abundance in symptomatic patients. *Eikenella* is a fastidious bacterium and a member of the HACEK group associated with infective endocarditis, periodontitis and also with ischemic stroke [28,29]. *Eikenella* lipopolysaccharides can trigger a pro-atherosclerotic endothelial response and enhance monocyte adhesion [30]. Interestingly, *Eikenella* abundance was lower in severe compared to moderate stenosis, suggesting a complex relationship between the microbiota load, degree of stenosis, plaque instability and symptoms/clinical events. In contrast to our findings, in a study of the oral microbiota in 20 patients with coronary artery disease, oral *Eikenella* was decreased in patients Compared to controls, and was linked to worse cardiovascular outcomes [28].

Previous research has identified oral bacterial species, including *Eikenella corrodens,* in both coronary and carotid plaques. A systematic review [31] of 63 studies covering 1791 coronary artery patients revealed *Eikenella* among a broader list of oral taxa found in plaques. Depending on method (16S rRNA and PCR based studies), ranging from 54.8 to 15.6%. Figuero [8] detected *Eikenella corrodens* in 54.8% of 46 carotid plaques by nested PCR with specific primers, while Mahendra [9] detected *Eikenella corrodens* in 27.5% of coronary atherosclerotic plaques in 51 coronary artery bypass patients with chronic periodontitis by PCR with specific primers. Kozarov [7] demonstrated the frequent detection of oral bacterial DNA in atheromas (29 patients) from multiple vascular sites using qPCR for ten species, including *Eikenella corrodens*, but the percentage was not given. Sato [32] investigated 54 carotid plaques by metagenomic 16S rRNA sequencing, but only five plaques were available for analysis. Bacterial DNA was much lower in carotid plaques (9.3%) compared to studies using PCR (50–90%).

To the best of our knowledge, we are the first to detect *Eikenella corrodens* in 100% of samples, with high detection rates for other bacteria (80–96.7%) compared to previous studies [7–9]. This might suggest a higher prevalence, but the discrepancy may also be partly explained by detection methods, sample sizes and patient populations. Quantitative RT-PCR shows higher sensitivity than conventional PCR for bacterial detection in human clinical samples [33], and together with specific primers and optimized sample handling, this is likely to increase the detection rate.

Ultimately, all though *Eikenella* is less studied than more common oral pathogens, such as *Fusobacterium* and *Porphyromonas,* previous evidence indicates its pro-atherogenic properties. Our findings suggest that *Eikenella* may play a role in plaque destabilization, all though further evidence is needed. Its universal detection in carotid plaques may indicate a more important role in atherosclerosis than currently recognized. Although our study was not designed to characterize a full microbial profile, the presence of multiple oral pathogens – rather than a single species – may be relevant in the context of atherosclerotic lesions. However, there was no correlation between the relative quantity of bacteria in the oral cavity and plaques. Therefore, the mechanism is more likely to involve systemic inflammatory pathways rather than direct dissemination of oral bacteria than through bacteremia. This study has several limitations. The sample size was modest, and we lacked detailed oral examinations and oral hygiene habits over time, as well as a detailed history of periodontal treatment or tooth loss, which we know may influence oral microbiota composition.

Also, a cross-sectional study cannot explain causality. The nature of the study precludes causal inference; therefore, the findings are only descriptive and must be interpreted with caution. However, all participants were investigated under standardized fasting conditions, and we had detailed information on risk factors, ultrasound, and blood and oral samples collected in the same manner. The consistency between oral swab samples and saliva results supports the robustness of our data. Moreover, the simultaneous investigation of oral and plaque samples using highly sensitive qRT-PCR detection provides novel insights into the link between the oral microbiota and carotid atherosclerosis.

## Conclusion

Patients with carotid atherosclerosis have a distinct oral microbiota profile characterized by lower alpha diversity, different beta diversity, and significantly different bacterial taxa abundance compared to healthy controls. We found higher oral relative abundance of the *Eikenella* genus in patients with recent cerebrovascular symptoms as compared to asymptomatic patients and, we detected the *Eikenella corrodens* species in all 30 carotid plaques. Our findings suggest that *Eikenella* may play a more important role in atherogenesis than previously thought and indicate a potential association between oral *Eikenella* and carotid plaque instability, although additional research is needed to determine whether it contributes directly and can be used as a biomarker. Further mechanistic and longitudinal studies are needed to better understand the role of *Eikenella* and other oral bacteria in inflammation, endothelial dysfunction and cerebrovascular risk.

## Supplementary Material

Supplementary_material_.docx Supplementary_material_.docx

## Data Availability

The data will be available upon request to the corresponding author.

## References

[1] Trøseid M, Andersen GØ, Broch K, et al. The gut microbiome in coronary artery disease and heart failure: Current knowledge and future directions. EBioMedicine. 2020 Feb;52:102649. doi: 10.1016/j.ebiom.2020.102649 32062353 PMC7016372

[2] Sun W, Huang S, Yang X, et al. The oral microbiome of patients with ischemic stroke predicts their severity and prognosis. Front Immunol. 2023;14:1171898. doi: 10.3389/fimmu.2023.1171898 37138888 PMC10150016

[3] Hajishengallis G, Chavakis T. Local and systemic mechanisms linking periodontal disease and inflammatory comorbidities. Nat Rev Immunol. 2021;21(7):426–440. doi: 10.1038/s41577-020-00488-6 33510490 PMC7841384

[4] Tonelli A, Lumngwena EN, Ntusi NAB. The oral microbiome in the pathophysiology of cardiovascular disease. Nat Rev Cardiol. 2023;20:386–403. doi: 10.1038/s41569-022-00825-3 36624275

[5] Akhi R, Lavrinienko A, Hakula M, et al. Oral microbiome diversity associates with carotid intima media thickness in middle-aged male subjects. Commun Med (Lond). 2025;5(1):66. doi: 10.1038/s43856-025-00773-2 40050445 PMC11885836

[6] Koren O, Spor A, Felin J, et al. Human oral, gut, and plaque microbiota in patients with atherosclerosis. Proc Natl Acad Sci U S A. 2011 Mar 15;108(Suppl 1):4592–4598. doi: 10.1073/pnas.1011383107 20937873 PMC3063583

[7] Kozarov E, Sweier D, Shelburne C, et al. Detection of bacterial DNA in atheromatous plaques by quantitative PCR. Microb Infect. 2006;8(3):687–693. doi: 10.1016/j.micinf.2005.09.004 16513386

[8] Figuero E, Sánchez-Beltrán M, Cuesta-Frechoso S, et al. Detection of periodontal bacteria in atheromatous plaque by nested polymerase chain reaction. J Periodontol. 2011;82(10):1469–1477. doi: 10.1902/jop.2011.100719 21453047

[9] Mahendra J, Mahendra L, Kurian VM, et al. Prevalence of periodontal pathogens in coronary atherosclerotic plaque of patients undergoing coronary artery bypass graft surgery. J Maxillofac Oral Surg. 2009;8(2):108–113. doi: 10.1007/s12663-009-0028-5 23139486 PMC3453943

[10] Peng X, Cheng L, You Y, et al. Oral microbiota in human systematic diseases. Int J Oral Sci. 2022 Mar 2;14(1):14. doi: 10.1038/s41368-022-00163-7 35236828 PMC8891310

[11] Zhang B, Khalaf H, Sirsjö A, et al. Gingipains from the periodontal pathogen porphyromonas gingivalis play a significant role in regulation of angiopoietin 1 and angiopoietin 2 in human aortic smooth muscle cells. Infect Immun. 2015;83(11):4256–4265. doi: 10.1128/IAI.00498-15 26283334 PMC4598411

[12] Gibson 3rd FC, Hong C, Chou HH, et al. Innate immune recognition of invasive bacteria accelerates atherosclerosis in apolipoprotein e-deficient mice. Circulation. 2004;109(22):2801–2806. doi: 10.1161/01.CIR.0000129769.17895.F0 15123526

[13] Li Y, Cui J, Liu Y, et al. Oral, tongue-coating microbiota, and metabolic disorders: a novel area of interactive research. Front Cardiovasc Med. 2021;8:730203. doi: 10.3389/fcvm.2021.730203 34490384 PMC8417575

[14] Pignatelli P, Fabietti G, Ricci A, et al. How periodontal disease and presence of nitric oxide reducing oral bacteria can affect blood pressure. Int J Mol Sci. 2020;21(20):7538. doi: 10.3390/ijms21207538 33066082 PMC7589924

[15] Stø K, Valeur J, Ueland T, et al. Fecal level of butyric acid, a microbiome-derived metabolite, is increased in patients with severe carotid atherosclerosis. Sci Rep. 2022 Dec 26;12(1):22378. doi: 10.1038/s41598-022-26759-x 36572703 PMC9792531

[16] Fadrosh DW, Ma B, Gajer P, et al. An improved dual‐indexing approach for multiplexed 16S rRNA gene sequencing on the Illumina MiSeq platform. Microbiome. 2014;2(1):6. doi: 10.1186/2049-2618-2-6 24558975 PMC3940169

[17] Bushnell B (accessed January 2024). https://jgi.doe.gov/data-and-tools/bbtools/

[18] Martin M. Cutadapt removes adapter sequences from high-throughput sequencing reads. EMBnet.journal. 2011;17(1):10–12. doi: 10.14806/ej.17.1.200

[19] Bushnell B, Rood J, Singer E. BBMerge – accurate paired shotgun read merging via overlap. PLoS One. 2017;12(10):e0185056. doi: 10.1371/journal.pone.0185056 29073143 PMC5657622

[20] Bolyen E, Rideout JR, Dillon MR, et al. Reproducible, interactive, scalable and extensible microbiome data science using QIIME 2. Nat Biotechnol. 2019;37:852–857. doi: 10.1038/s41587-019-0209-9 31341288 PMC7015180

[21] Quast C, Pruesse E, Yilmaz P, et al. The SILVA ribosomal RNA gene database project: improved data processing and web-based tools. Nucl Acids Res. 2013;41(D1):D590–D596. doi: 10.1093/nar/gks1219 23193283 PMC3531112

[22] Lin H, Peddada SD. Analysis of compositions of microbiomes with bias correction. Nat Commun. 2020;11:3514. doi: 10.1038/s41467-020-17041-7 32665548 PMC7360769

[23] Braga RRR, Carvalho MAR, Bruña-Romero O, et al. Quantification of five putative periodontal pathogens in female patients with and without chronic periodontitis by real-time polymerase chain reaction. Anaerobe. 2010 Jun;16(3):234–239. doi: 10.1016/j.anaerobe.2010.02.007 20193770

[24] Fåk F, Tremaroli V, Bergström G, et al. Oral microbiota in patients with atherosclerosis. Atherosclerosis. 2015 Dec;243(2):573–578. doi: 10.1016/j.atherosclerosis.2015.10.097 26536303

[25] Wang Y, Hou J, Tsui JC, et al. Unique gut microbiome signatures among adult patients with moderate to severe atopic dermatitis in Southern Chinese. Int J Mol Sci. 2023 Aug 16;24(16):12856. doi: 10.3390/ijms241612856 37629036 PMC10454836

[26] Boaden E, Lyons M, Singhrao SK, et al. Oral flora in acute stroke patients: a prospective exploratory observational study. Gerodontology. 2017;34:343–356. doi: 10.1111/ger.12271 28543778

[27] Kato-Kogoe N, Sakaguchi S, Kamiya K, et al. Characterization of salivary microbiota in patients with atherosclerotic cardiovascular disease: a case-control study. J Atheroscler Thromb. 2022 Mar 1;29(3):403–421. doi: 10.5551/jat.60608 33612553 PMC8894113

[28] Bouzid F, Gtif I, Alfadhli S, et al. A potential oral microbiome signature associated with coronary artery disease in Tunisia. Biosci Rep. 2022;42(7):BSR20220583. doi: 10.1042/BSR20220583 35695679 PMC9251586

[29] Socransky SS, Haffajee AD, Cugini MA, et al. Microbial complexes in subgingival plaque. J Clin Periodontol. 1998;25(2):134–144. doi: 10.1111/j.1600-051X.1998.tb02419.x 9495612

[30] Viafara-Garcia SM, Gualtero DF, Avila-Ceballos D, et al. Eikenella corrodens lipopolysaccharide stimulates the pro-atherosclerotic response in human coronary artery endothelial cells and monocyte adhesion. Eur J Oral Sci. 2018 Dec;126(6):476–484. doi: 10.1111/eos.12580 30357941

[31] Chhibber-Goel J, Singhal V, Bhowmik D, et al. Linkages between oral commensal bacteria and atherosclerotic plaques in coronary artery disease patients. npj Biofilms Microbiomes. 2016;2:7. doi: 10.1038/s41522-016-0009-7 28649401 PMC5460270

[32] Sato A, Arai S, Sumi K, et al. Metagenomic analysis of bacterial microflora in dental and atherosclerotic plaques of patients with internal carotid artery stenosis. Clin Med Insights Cardiol. 2024;18:11795468231225852. doi: 10.1177/11795468231225852 38328472 PMC10848802

[33] Topcuoglu N, Paltura C, Kulekci M, et al. Real-time polymerase chain reaction versus conventional PCR: a comparison between two methods for the detection of *Fusobacterium Nucleatum* in saliva, nasopharyngeal secretion and middle ear effusion samples. Biotechnol Biotechnol Equipment. 2013;27(3):3825–3828. doi: 10.5504/BBEQ.2013.0022

