# Supplementary material for: Oral Eikenella as a potential new biomarker of symptomatic carotid atherosclerosis
Source: J Oral Microbiol. 2026 Jan 12;18(1):2613521. doi: 10.1080/20002297.2026.2613521 (PMC12798661; doi:10.1080/20002297.2026.2613521)
Supplement: Supplementary_material_.docx [file ZJOM_A_2613521_SM0516.docx]

**Supplementary Material**

**Supplementary Figure 1**

Flowchart of patients and controls.

A total of 78 participants were included in the oral 16s rRNA analysis, including 41 patients (15 symptomatic and 26 asymptomatic) and 37 healthy controls, and 30 patients provided carotid plaques and oral swab samples available for qPCR analysis


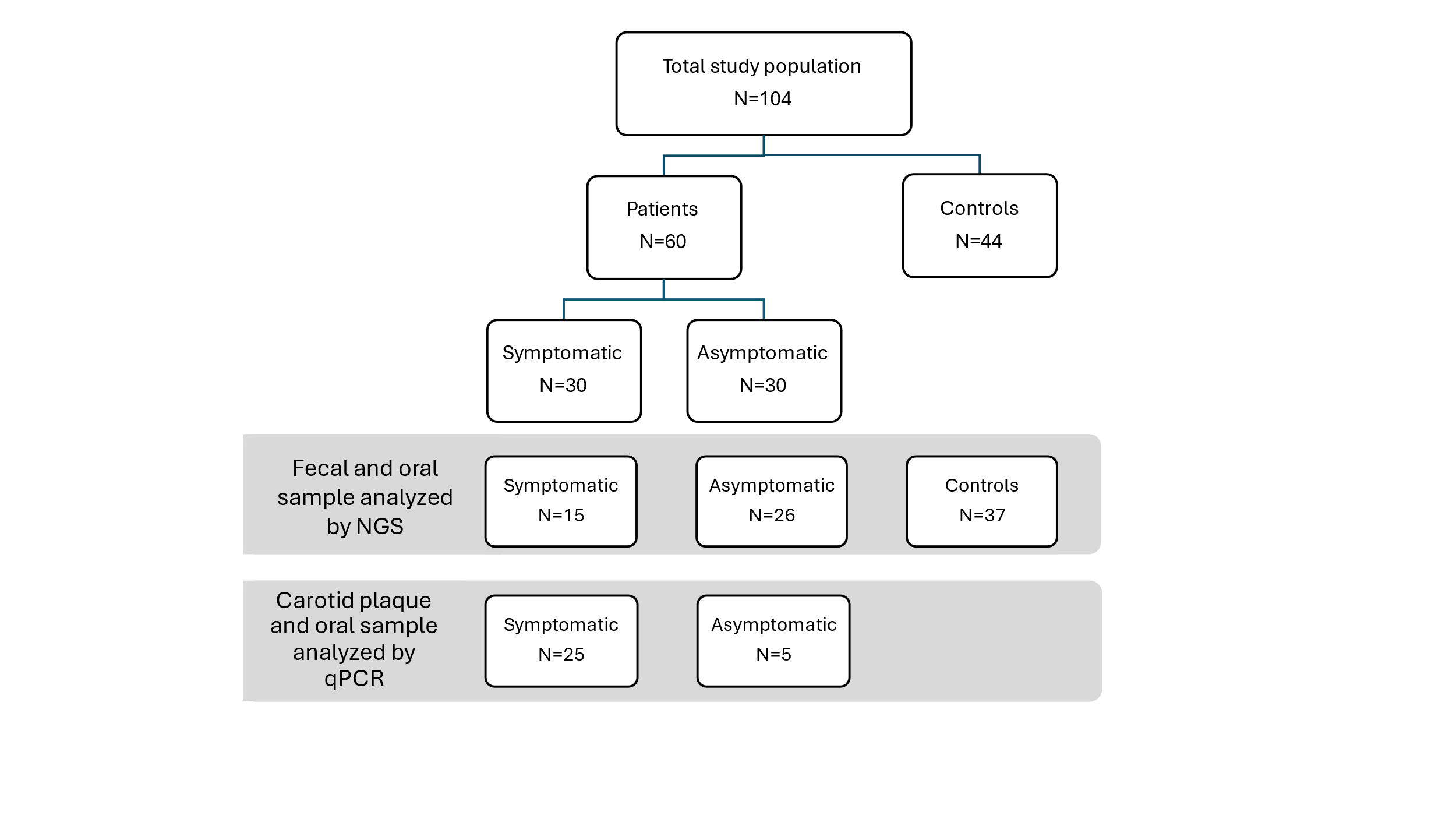


**Supplementary figure 2 Oral microbiota in saliva samples in patients versus healthy controls.**

Saliva shows the same patterns for beta and alpha diversities as oral swabs. Beta diversity (Jaccard-distances) show significant differences (A), and Alpha diversity (Observed features) is significantly lower in patients (B).

*
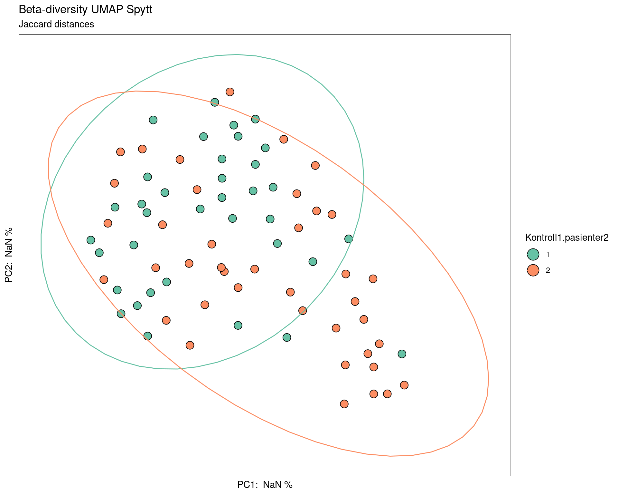
*
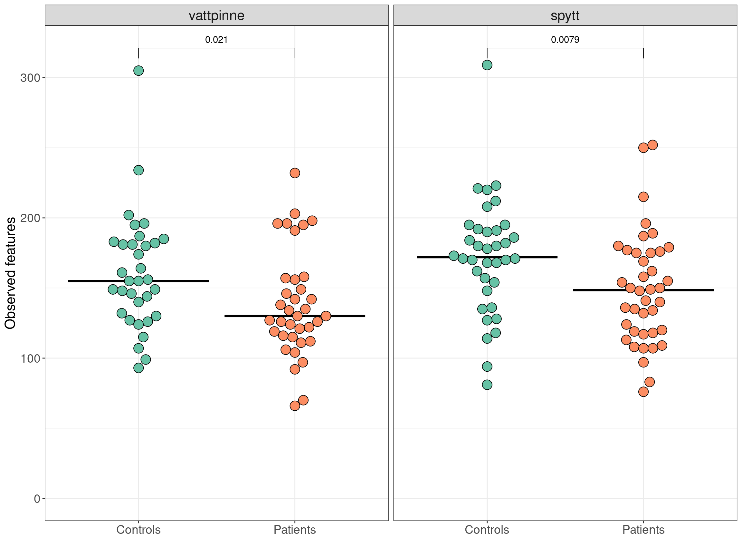


1. (B)

*p=*0.008

*p=*0.002

(2a)

umap2

*p*=0.008

(2b)

umap1

**Supplementary Table 1**

Up- and downregulated bacterial genera in patients versus controls (oral swabs, q<0.1, log2FC>

**Supplementarty table 1:** Organism-specific primers for qRT-PCR detection
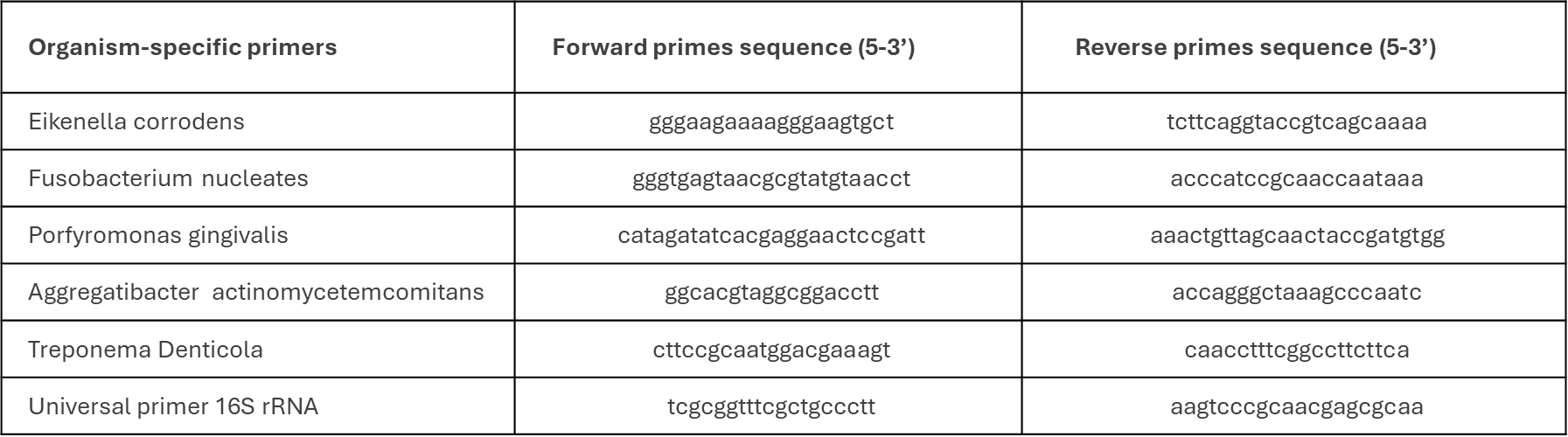


**Supplementary table 2:**

Up- and downregulated bacterial genera in all patients versus healthy controls (oral swabs, q<0.1, log2FC>1)

| **Taxon – genus level** |  |
| --- | --- |
| Firmicutes;c__Bacilli;o__Lactobacillales;f__Lactobacillaceae;g__Lactobacillus | upregulated |
| Actinobacteriota;c__Actinobacteria;o__Bifidobacteriales;f__Bifidobacteriaceae;g__Scardovia | upregulated |
| Proteobacteria;c__Gammaproteobacteria;o__Burkholderiales;f__Neisseriaceae;g__Bergeriella | downregulated |
| Actinobacteriota;c__Coriobacteriia;o__Coriobacteriales;f__Eggerthellaceae;g__Cryptobacterium | upregulated |
| Firmicutes;c__Bacilli;o__Staphylococcales;f__Staphylococcaceae;g__Staphylococcus | upregulated |
| Firmicutes;c__Bacilli;o__Mycoplasmatales;f__Mycoplasmataceae;g__Mycoplasma | downregulated |
| Firmicutes;c__Bacilli;o__Lactobacillales;f__Lactobacillaceae;__ | upregulated |
| Firmicutes;c__Bacilli;o__Lactobacillales;f__Lactobacillaceae;g__ | upregulated |
| Firmicutes;c__Clostridia;o__Peptostreptococcales-Tissierellales;f__Peptostreptococcaceae;g__Filifactor | upregulated |
| Actinobacteriota;c__Actinobacteria;o__Propionibacteriales;f__Propionibacteriaceae;g__Propionibacterium | upregulated |
| Bacteroidota;c__Bacteroidia;o__Bacteroidales;f__Paludibacteraceae;g__F0058 | upregulated |
| Firmicutes;c__Clostridia;o__Peptostreptococcales-Tissierellales;f__Anaerovoracaceae;g__Family_XIII_UCG-001 | downregulated |
| Actinobacteriota;c__Actinobacteria;o**Micrococcales;**;__ | upregulated |
| Firmicutes;c__Negativicutes;o__Veillonellales-Selenomonadales;f__Selenomonadaceae;g__uncultured | upregulated |
| Proteobacteria;c__Gammaproteobacteria;o__Pseudomonadales;f__Pseudomonadaceae;g__Pseudomonas | upregulated |
| Patescibacteria;c__Gracilibacteria;o__JGI_0000069-P22;f__JGI_0000069-P22;g__JGI_0000069-P22 | downregulated |
| Proteobacteria;c__Gammaproteobacteria;o__Burkholderiales;f__Alcaligenaceae;__ | downregulated |
| Firmicutes;c__Clostridia;o__Lachnospirales;f__Lachnospiraceae;g__Johnsonella | downregulated |
| Bacteroidota;c__Bacteroidia;o__Bacteroidales;f__Prevotellaceae;g__Alloprevotella | downregulated |
| Proteobacteria;c__Gammaproteobacteria;o__Burkholderiales;f__Neisseriaceae;g__Neisseria | downregulated |
| Firmicutes;c__Negativicutes;o__Veillonellales-Selenomonadales;f__Selenomonadaceae;g__Selenomonadaceae | downregulated |
| Firmicutes;c__Negativicutes;o__Veillonellales-Selenomonadales;f__Selenomonadaceae;g__Selenomonas | downregulated |

**Supplementary table 3**

Up- and downregulated bacterial genera in symptomatic patients versus asymptomatic patients (oral swabs, q<0.1, log2FC>1)

| **Taxon** |  |
| --- | --- |
| Patescibacteria;c__Saccharimonadia;o__Saccharimonadales;f__Saccharimonadaceae;g__Saccharimonadaceae | upregulated |
| Firmicutes;c__Bacilli;o__Lactobacillales;f__Aerococcaceae;g__Abiotrophia 1.720936 | upregulated |
| Proteobacteria;c__Gammaproteobacteria;o__Burkholderiales;f__Burkholderiaceae;g__Lautropia | upregulated |
| Proteobacteria;c__Gammaproteobacteria;o__Enterobacterales;f__Pasteurellaceae;g__Actinobacillus | upregulated |
| Proteobacteria;c__Gammaproteobacteria;o__Burkholderiales;f__Neisseriaceae;g__Eikenella | upregulated |

**Supplementary table 4**

Correlations for demographics and inflammatory markers, alpha diversity and dysbiosis indices in patients and controls.

|  | Patients  N=41 | Dysbiosis index oral swab | Alpha diversity oral swab | Controls  N=37 | Dysbiosis index oral swab | Alpha diversity oral swab |
| --- | --- | --- | --- | --- | --- | --- |
| Age, (years) | 72 (6)** | -0.22 | 0.04 | 67 (8) | -0.02 | -0.13 |
| Male sex, n (%) | 18 (44) | 0.01 | 0.30 | 10 (27) | 0.24 | 0.18 |
| Body Mass Index (kg/m^2^) | 25.5 (3.8) | -0.01 | 0.20 | 25.1 (3.2) | 0.09 | -0.07 |
| Waist-hip ratio (cm/cm) | 0.95 (0.08)** | -0.42* | 0.23 | 0.89 (0.07) | 0.16 | 0.04 |
| Current smoker | 5 (12) | 0.22 | -0.37* | 2 (5) | 0.17 | -0.09 |
| Hypertension | 32 (78)*** | -0.26 | 0.19 | 8 (22) | 0.00 | -0.19 |
| Type 2 Diabetes mellitus | 10 (24)*** | 0.07 | -0.12 | 0 (0) | . | . |
| Hypercholesterolemia | 23 (56)** | 0.06 | 0.01 | 7 (19) | 0.02 | -0.40* |
| Anti-platelet treatment | 33 (81)*** | 0.03 | 0.15 | 3 (8) | -0.23 | -0.22 |
| Statin treatment | 32 (78)*** | 0.25 | -0.08 | 4 (11) | 0.05 | -0.36* |
| C-reactive protein, (mg/L) | 1.1 (0.7, 2.5)* | 0.07 | 0.07 | 0.9 (0.5, 1.6) | 0.27 | 0.21 |
| Leukocyte count, (10^9^/L) | 7.8 (1.9)*** | -0.01 | 0.04 | 5.1 (1.1) | 0.16 | -0.11 |
| Total cholesterol, (mM) | 4.2 (1.0)*** | 0.23 | -0.15 | 5.3 (0.9) | -0.11 | 0.35* |
| LDL cholesterol, (mM) | 2.3 (0.9)*** | -0.01 | 0.03 | 3.2 (0.8) | -0.06 | 0.37* |
| HbA1c (%)* | 5.7 (0.9)** | -0.23 | 0.37 | 5.3 (0.3) | 0.15 | -0.01 |
| Antibiotics last 3 months | 8 (20)** | 0.08 | -0.27 | 0 (0) | . | . |

Continuous data are given as mean (SD) except CRP which is given as median (IQR) while categorical data are shown as n (%).

For comparison patients and controls: *p<0.05, **p<0.01, ***p<0.001. For correlations: *p<0.05, **p<0.01

**Additional Supplementary material:**

A dysbiosis index for oral swab samples for carotid atherosclerosis based on the up- and down regulated bacteria was calculated as followed: log((d__Bacteria;p__Firmicutes;c__Bacilli;o__Lactobacillales;f__Lactobacillaceae;g__Lactobacillus+ d__Bacteria;p__Actinobacteriota;c__Actinobacteria;o__Bifidobacteriales;f__Bifidobacteriaceae;g__Scardovia+ d__Bacteria;p__Actinobacteriota;c__Coriobacteriia;o__Coriobacteriales;f__Eggerthellaceae;g__Cryptobacterium+ d__Bacteria;p__Firmicutes;c__Bacilli;o__Staphylococcales;f__Staphylococcaceae;g__Staphylococcus+ d__Bacteria;p__Firmicutes;c__Bacilli;o__Lactobacillales;f__Lactobacillaceae;__+ d__Bacteria;p__Firmicutes;c__Bacilli;o__Lactobacillales;f__Lactobacillaceae;g__+ d__Bacteria;p__Firmicutes;c__Clostridia;o__Peptostreptococcales-
